# Supplementary material for: Lenalidomide Efficacy in Patients with MDS and Del-5q: Real-World Data from the Hellenic (Greek) National Myelodysplastic & Hypoplastic Syndromes Registry (EAKMYS)
Source: Cancers (Basel). 2025 Apr 22;17(9):1388. doi: 10.3390/cancers17091388 (PMC12071050; doi:10.3390/cancers17091388)
Supplement: Supplementary file 1 [file cancers-17-01388-s001.zip › cancers-3529631-supplementary.pdf]

Supplementary material

Table S1. Most common recurrent cytogenetic abnormalities observed in additional to Del(5)q (Groups III and IV)

**Abnormality**	**N**	**Percentage among Groups III and IV**	**Percentage overall**
Trisomy 8	14	27.4	5.9
Monosomy 7 / Del(7)q	6	11.8	2.5
Del(20)(q11;q13)	5	9.8	2.1
Monosomy 18	4	7.8	1.7
Trisomy 21	3	5.9	1.3
Del(13)(q12;q14)	3	5.9	1.3
Trisomy 11	2	3.9	0.8
Monosomy 9, 13, 17 or 20	2	3.9	0.8
Del(1)(p22;p36)	2	3.9	0.8
add(1p36)	2	3.9	0.8
add(6)(p21)	2	3.9	0.8
add12(p13)	2	3.9	0.8
markers chromosomes	16	31.4	6.7

Abbreviation: Del(5q), isolated deletion of chromosome 5q.

Table S2. Proportions of patients who achieved normalization of hematological parameters post-lenalidomide treatment (IWG criteria)

		Normal Hb values	Normal ANC values	Normal PLT values
		**n (%)**	**n (%)**	**n (%)**
Group-I – OR	n = 129	101 (78.3)	101 (78.3)	90 (69.8)
Group-I – MR	n = 114	100 (87.7)	94 (82.4)	84 (73.7)
Group-I – MiR/HI	n = 15	1 (6.7)	7 (46.7)	6 (40.0)
Group-II – OR	n = 25	17 (68.0)	18 (72.0)	15 (60.0)
Group-II – MR	n = 18	15 (83.3)	16 (88.9)	12 (66.7)
Group-II – MiR/HI	n = 7	2 (28.6)	2 (28.6)	3 (42.8)
Group-III – OR	n = 16	11 (68.8)	10 (62.5)	7 (43.8)
Group-III – MR	n = 10	10 (100.0)	7 (70.0)	5 (50.0)
Group-III – MiR/HI	n = 6	1 (16.7)	3 (50.0)	2 (33.3)
Group-IV – OR	n = 7	4 (57.1)	5 (71.4)	2 (28.6)
Group-IV – MR	n = 4	4 (100.0)	3 (75.0)	2 (50.0)
Group-IV – MiR/HI	n = 3	0 (0.0)	2 (66.7)	0 (0.0)
Total – OR	n = 177	133 (75.1)	134 (75.7)	114 (64.4)
Total – MR	n = 146	129 (88.3)	120 (82.2)	103 (70.5)
Total – PR/HI	n = 31	4 (12.9)	14 (45.2)	11 (35.5)

Abbreviations: ANC, absolute neutrophil count; CR, complete response; Hb, hemoglobin; HI, hematological improvement; IWG, International Working Group; OR, overall response; PR, partial response; PLT, platelet.

Normal values: Hb >11.5 for females and >12.0 for males, ANC > 1.5 x 10^9/μL, PLT: 140-399 x 10^9/μL

Table S3. Duration of response to treatment in relation to group, type of response and excess of BM blasts

	**Duration of response (Median K–M estimates)**	**95% CI**	***P* Values (Mantel–Cox)**
Group-I	34.0	26.0-42.0	> 0.050
Group-II	17.1	7.9-26.3	
Group-III	45.6	15.2-76.0	
Group-IV	22.0	0.0-59.2	
MR	34.7	29.2-40.2	**< 0.001**
MiR	16.4	0.4-32.4	
BM blasts no excess	34.7	29.1-40.3	**= 0.022**
BM blasts excess	18.4	7.6-29.2	
Total	32.1	24.0-40.2	

Abbreviations: BM, bone marrow; CI, confidence interval; CR, complete response; KM, Kaplan–Meier; PR, partial response.

Table S4. Overall response to treatment, in relation to baseline features among evaluable patients (multivariate logistic regression model)

	Overall response to treatment					
	Nagelkerke R^2 = 0.17					
**Predictors**	**None**	**Partial**	**Complete**	**OR**	**95% CI**	***P* Values**
MCV at baseline	41		146	1.05	1.02-1.09	***0.002***
BM blasts (%) at baseline	41		146	0.86	0.79-0.93	***<0.001***
Transfusion dependency at baseline (Yes)	32		92	1.00		
Transfusion dependency at baseline (No)	9		54	1.87	0.79-4.43	*0.158*
MCV at baseline	41	31		1.04	0.99-1.08	*0.085*
BM blasts (%) at baseline	41	31		0.95	0.87-1.04	*0.268*
Transfusion dependency at baseline (Yes)	32	24		1.00		
Transfusion dependency at baseline (No)	9	7		0.95	0.30-2.97	*0.931*

Abbreviations: BM, bone marrow; CI, confidence interval; MCV, mean corpuscular volume; OR, odds ratio.
